# Induction immune-checkpoint inhibitors for resectable oncogene-mutant NSCLC: A multicenter pooled analysis

**DOI:** 10.1038/s41698-022-00301-8

**Published:** 2022-09-19

**Authors:** Chao Zhang, Hua-Fei Chen, Shi Yan, Lin Wu, Li-Xu Yan, Xiao-Long Yan, Dong-Sheng Yue, Chun-Wei Xu, Min Zheng, Ji-Sheng Li, Si-Yang Liu, Ling-Ling Yang, Ben-Yuan Jiang, Qiu-Xiang Ou, Zhen-Bin Qiu, Yang Shao, Yi-Long Wu, Wen-Zhao Zhong

**Affiliations:** 1 Guangdong Lung Cancer Institute, Guangdong Provincial Key Laboratory of Translational Medicine in Lung Cancer, Guangdong Provincial People’s Hospital & Guangdong Academy of Medical Sciences, Guangzhou, 510080 China; 2 School of Medicine, South China University of Technology, Guangzhou, 510006 China; 3 Department of Thoracic Disease Center, Zhejiang RongJun Hospital, Jiaxing, Zhejiang 314000 China; 4 Department of Thoracic Surgery II, Peking University Cancer Hospital and Institute, Beijing, 100142 China; 5 Department of Oncology, Hu Nan Provincial Tumor Hospital, Changsha, 410006 China; 6 Department of Pathology, Guangdong Provincial People’s Hospital & Guangdong Academy of Medical Sciences, Guangzhou, 510080 China; 7 Division of Thoracic Surgery, Tang Du Hospital of Fourth Military Medical University, Xi’an, Shanxi 710032 China; 8 Department of Lung Cancer, Tianjin Lung Cancer Center, Tianjin Cancer Institute and Hospital, Tianjin Medical University, Hexi, Tianjin, 300060 China; 9 Department of Respiratory Medicine, Jinling Hospital, Nanjing University School of Medicine, 305 Zhongshan Road, Nanjing, 210002 China; 10 Department of Thoracic Surgery, Affiliated Hospital of Guilin Medical University, Guilin, 541001 China; 11 Department of Chemotherapy, Cancer Center, Qilu Hospital, Shandong University, Jinan, 250012 China; 12 Geneseeq Research Institute, Geneseeq Technology Inc., Nanjing, 210032 China

**Keywords:** Non-small-cell lung cancer, Translational research, Surgical oncology

## Abstract

Despite limited efficacy of immunotherapy for advanced non-small-cell lung cancer (NSCLC) with driver mutations, whether neoadjuvant immunotherapy could be clinically valuable in those patients warrants further investigation. We utilized 40 oncogene-mutant NSCLC treated with induction immunotherapy from a large consecutive multicenter cohort. Overall response rate was 62.5% while 2 patients had disease progression. Of 39 patients that received surgery, R0 resection rate was 97.4%. The major pathological response (MPR) rate was 37.5% and the pathological complete response (pCR) rate was 12.5%. Pre-treatment PD-L1 expression was not a predictive biomarker in these patients. Median disease-free survival for all oncogenic mutation and EGFR mutation was 28.5 months. Indirect comparison through integrating CTONG1103 cohort showed neoadjuvant immunotherapy plus chemotherapy yielded the most superior efficacy among erlotinib and chemotherapy for resectable EGFR-mutant NSCLC. No MPR patients were identified with neoadjuvant immunotherapy plus chemotherapy for uncommon EGFR insertion or point mutations. Our results indicated the potential clinical feasibility of neoadjuvant immunotherapy for resectable localized oncogene-mutant NSCLC especially for EGFR-mutant NSCLC.

## Introduction

Anti-PD-1/PD-L1 antibodies, as immune checkpoint blockade therapy (ICB), have been widely applied in advanced lung cancer as a single agent, combined with other checkpoint blockade or platinum-based chemotherapy^1,2^. Such novel treatment could enhance antitumor immunity by restoring T lymphocytes’ function leading to long-term benefits^3^. However, for advanced non-small cell lung cancers (NSCLCs) harboring driver mutations such as *EGFR* or *ALK* mutations, immunotherapy exhibited impaired response compared to corresponding targeted therapies^4–6^. Even for those rare driver mutations, the efficacy of immunotherapy varied among different mutation types and clinicopathological status such as smoking and tumor mutation burden (TMB), showing significantly inferior response rate to targeted therapies^7^. The mechanism that caused impaired efficacy in these populations included limited infiltration of CD8^+^ T lymphocytes, lower PD-L1 expression and strong oncogenic pathway activation^8,9^. Unlike immunotherapy alone, IMpower 150 is the first trial that has demonstrated comparable efficacy as an immunotherapy-based combination strategy in pretreated *EGFR*/*ALK* mutant NSCLCs^10^. However, the response mechanism of immunotherapy in mutant NSCLC remained controversial.

For resectable NSCLCs, chemotherapy has remained as the standard neoadjuvant treatment for decades^11–13^. In recent years, multiple phase 2 trials of neoadjuvant immunotherapy have shown dramatic pathological response and elevated surgical resection rate^14–16^. However, data on oncogene-mutant NSCLC using novel neoadjuvant modalities are limited. CTONG1103 (EMERGING) (NCT01407822) study compared neoadjuvant targeted therapy and chemotherapy head-to-head and failed to show neither significantly improved major pathological response (MPR) nor overall survival (OS)^17^. This might be attributed to the highly selective therapeutic mechanism of targeted therapy, intratumor heterogeneity and concomitant mutations which leads to incomplete antitumor response^18,19^. In a previous study, neoadjuvant atezolizumab and chemotherapy showed dramatic pathological response even in subjects with sensitive *EGFR* mutations, providing the clinical potentials of immunotherapy-based combination strategy for oncogene-mutant NSCLC^16^.

Despite the limited efficacy of immunotherapy in advanced mutant NSCLC, little is known about whether neoadjuvant immunotherapy could be clinically valuable for NSCLC positive of driver mutations. Herein, we describe the clinical outcome and safety profile of neoadjuvant immunotherapy followed by surgery in a multi-center cohort of patients with oncogene-mutant localized NSCLC. We also conducted dynamic multi-omics sequencing to provide insight of the potential mechanisms that led to differential response of neoadjuvant immunotherapy.

## Results

### Patient characteristics

In total, patients treated with neoadjuvant immunotherapy were preliminarily screened from 26 centers around China, and 40 NSCLC patients that were treated with neoadjuvant immunotherapy and harboring EGFR mutations were collected from 8 thoracic and cancer centers. 60.0% (24/40) were men and 57.5% (23/40) were non-smokers. 32.5% (13/40) patients were pathologically or radiologically confirmed as N2 disease. Only one patient was suspected to have oligometastatic disease of left adrenal gland before neoadjuvant treatment which disappear after neoadjuvant immunotherapy. Among all, there were 47.5% (19/40) patients harboring *EGFR* mutation including exon 19del, exon 21L858R, exon 17–25 and exon 20 insertion. Other oncogene mutation included *KRAS*, *RET* fusion, *ALK* fusion, *ROS1* fusion, *BRAF* and *HER2* insertion. Percentage of residual tumor cells were evaluated in 87.5% (35/40) patients while the other patients did not undergo surgery or comprehensive pathological assessments to determine the specific values of their residual tumors. Clinical information was summarized in Table 1 regarding MPR status. Detailed demographics including therapeutic regimens, genomic profiles, physicians’ consideration for neoadjuvant immunotherapy and adverse events (AE) during neoadjuvant treatment, are shown in Supplementary Table 1.

**Table 1:** Clincal demograchics of enrolled patients regarding MPR status.

Characteristics	All patients (*N* = 40)	Patients with major pathological response (*N* = 15)	Patients without major pathological response (*N* = 25)	*P*-value
Age —— yrs				0.812
Mean ± SD	58.7 ± 10.9	58.6 ± 9.9	58.7 ± 11.5	
Median (range)	61 (25–75)	60 (36–72)	62 (25–75)	
Gender —— no. (%)				0.740
Female	16 (40.0)	5 (28.6)	11 (44.0)	
Male	24 (60.0)	10 (71.4)	14 (56.0)	
Smoking status —— no. (%)				0.749
Never	23 (57.5)	8 (53.3)	15 (60.0)	
Former/current	17 (42.5)	7 (46.6)	10 (40.0)	
Clinical stage —— no. (%)				0.580
II	10 (25.0)	5 (35.7)	5 (20.0)	
IIIA	22 (55.0)	7 (50.0)	15 (60.0)	
IIIB-IVA	8 (20.0)*	3 (14.3)	5 (20.0)	
Mutation features —— no. (%)				0.587
EGFR alteration	19 (47.5)	8 (50.0)	11 (44.0)	
KRAS alteration	9 (22.5)	4 (28.6)	5 (20.0)	
Other mutations	12 (30.0)	3 (21.4)	9 (36.0)	
PD-L1 expression——no. (%)				0.208
Negative	9 (22.5)	4 (26.7)	5 (20.0)	
1–49%	7 (17.5)	2 (13.3)	5 (20.0)	
≥50%	15 (37.5)	8 (53.3)	7 (28.0)	
Unknown	9 (22.5)	1 (6.7)	8 (32.0)	

^*^One patient with suspected left adrenal gland metastatsis was included which dismissed after neoadjuvant immunotherapy plus chemotherapy.

### Clinical efficacy and safety profile of all oncogenic drivers

**Fig. 1:**
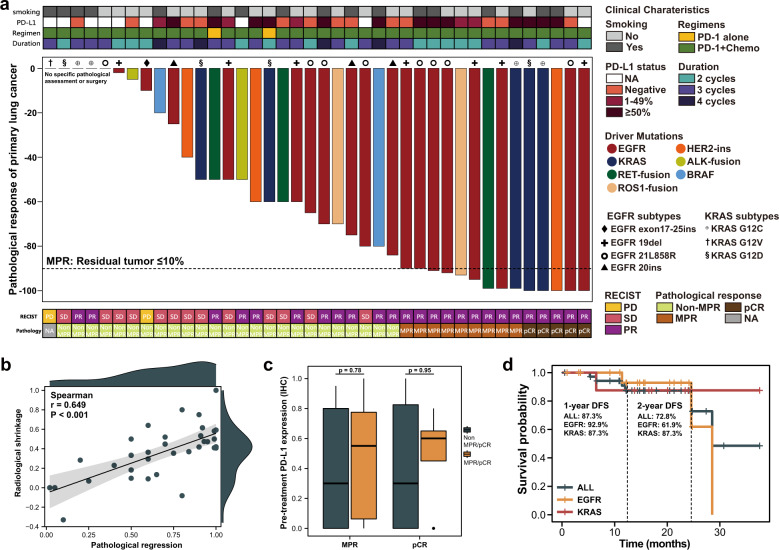
Overview of clinicopathological characteristics and clinical outcome of oncogene-mutant NSCLC treated with neoadjuvant immunotherapy. **a** Overview of clinicopathological charteristics and clinical outcome of oncogene-mutant NSCLC treated with neoadjuvant immunotherapy. Clinicopathological charateristics including smoking status, therpeutic regimens, PD-L1 expression, treatment duration and preoperative radiologic response (according to Response Evaluation Criteria in Solid Tumors [RECIST]) were annotated for each patients. The horizontal dash line indicates the threshold for a major pathological response (90% regression). Pathological regression was presented in colored bar plot regarding different oncogene mutation. **b** Correlation between radiological shrinkage and pathological regression. Spearman correlation was used to measure the relevance. **c** Association between PD-L1 expression and MPR status. Centre line represents the median value of PD-L1 expression. Error bars represent the upper and lower quartiles of PD-L1 expression and whiskers defines the minimal and maximun value. PD Progression disease, SD Stable disease, PR Partial response, MPR Major pathological response, pCR Pathological complete response. **d** Disease-free survival (DFS) of all patients, EGFR-mutant patients and KRAS-mutant patients treated with neoadjuvant immunotherapy through Kaplan-Meier survival analysis.

All patients have completed neoadjuvant immunotherapy without treatment discontinuation owing to severe toxicity. No new safety signals were identified regarding previous studies. Only two patients had grade 3 anemia, while others exhibited grade 1-2 adverse events (AEs) such as rash and diarrhea. Detailed AEs profile are shown in Supplementary Table 1. Overall, 62.5% (25/40) patients achieved partial response (PR) while 5.0% (2/40) had disease progression after completion of neoadjuvant immunotherapy. The N2 downstaging rate was 60.0% (9/15) for patients with pathologically or radiologically confirmed N2 disease. Of the two patients with progressive disease, one patient did not receive surgery due to newly-developed metastatic disease, while the other patient’s tumor was still considered to be resectable based on multidisciplinary discussion. Among the patients who underwent surgical resection, only one patient was reported with R2 resection while others achieved R0 resection. The detailed clinicopathological features of enrolled patients upon radiological and pathological response were presented in Fig. 1a. For patients treated with neoadjuvant immunotherapy, 37.5% (15/40) achieved MPR and 12.5% (5/40) achieved pCR.

We further assessed the correlation between radiological shrinkage and pathological regression of which a strong correlation was found (*p*-value < 0.001) probably due to most patients (91.2%) receiving neoadjuvant immunotherapy and chemotherapy (Fig. 1b). No significant associations of PD-L1 level were found between MPR and non-MPR patients (Fig. 1c). With a median follow-up time of 15.5 months, median disease-free survival (DFS) for both all oncogenic mutation and EGFR mutation were 28.5 months while median DFS for KRAS mutation was not reached. 1-year DFS for all oncogenic mutation, EGFR mutation and KRAS mutation was 87.3%, 92.9%, 87.3%, and 72.8%, 61.9%, 87.3% for 2-year DFS, respectively (Fig. 1d). Regarding MPR status, patients achieved MPR in both groups showed favorable trending of DFS (Supplementary Fig. 1).

### Comparison of different treatment modalities for localized EGFR-mutant NSCLC

**Fig. 2:**
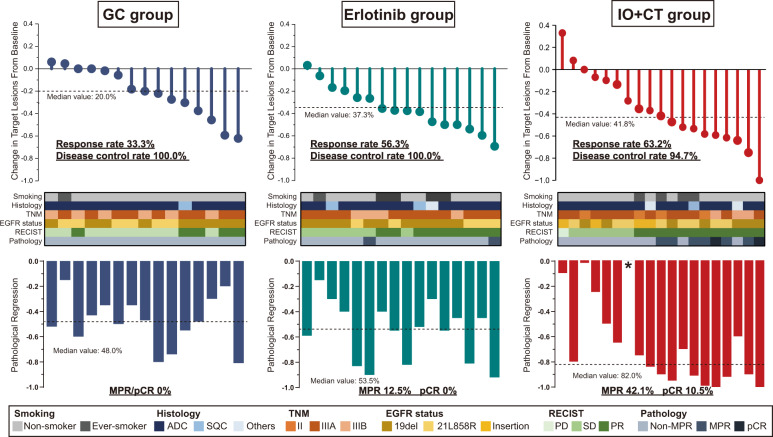
Efficacy of neoadjuvant treatments for EGFR-mutant NSCLC. Comparison of different treatment modalities for resectable EGFR-mutant NSCLC in regard to radiological response and pathological assessment by integrating chemotherapy and EGFR-TKI cohorts from CTONG1103 study. ADC Adenocarcinoma, SQC Squamous cell carcinoma, SD Stable disease, PR Partial response, PD Progression disease, CR Compelete response, MPR Major pathological response, pCR P athological complete response.

As the most common driver mutation in lung cancer, we emphatically analyzed the subgroup of EGFR-mutant NSCLC. We integrated the individual data of EGFR-mutant cohort from CTONG1103 (NCT01407822) study to compare the efficacy of different neoadjuvant treatment modalities. Patients were stratified into 3 groups for subsequent analysis: chemotherapy (GC regimen), targeted therapy (erlotinib) and immunotherapy plus chemotherapy (IO + CT). The baseline characteristics were relatively well balanced among groups in terms of smoking status, histology and EGFR mutation subtypes. EGFR exon 21 L858R was the most common subtype among groups (Table 2). In GC group, the response rate was 33.3% (5/15) and 56.3% for erlotinib group while elevated response rate of 63.2% was identified in IO + CT group. Of note, disease control rate (DCR) was 100% for both GC and erlotinib group, and one patient (5.3%) in IO + CT group had disease progress after neoadjuvant immunotherapy plus chemotherapy (Fig. 2). However, this patient was considered for radical resection after multidisplenary discussion and received surgery successfully. No sever postoperative complication was found except for one patient who had blood transfusion in regard to Clavien-Dindo score. Detailed clinicopathological features were summarized in Table 2. Only one patient in IO + CT group did not have specific value of residual viable tumor. The MPR in IO + CT group was significantly higher than erlotinib group (42.1% vs. 12.5%, odds ratio, 5.09; 95%CI, 1.04-26.39; *P* = 0.071) (Supplementary Fig. 2). Strikingly, while no pCR was found in both GC and erlotinib group, neoadjuvant immunotherapy plus chemotherapy yielded 10.5% pCR (odds ratio, not available; *P* = 0.489) (Supplementary Fig. 2). Among these patients, 66.7% (4/6) patients with EGFR exon19del achieved MPR and 16.7% (1/6) achieved pCR. On the other hand, 50.0% (4/8) patients of EGFR exon21 L858R had MPR and 12.5% (1/8) achieved pCR. Six patients detected with rare EGFR mutations, including insertions (3 EGFR 20ins, 1 EGFR exon17-25ins and 1 EGFR exon19ins) and rare point mutation (EGFR exon21 L861Q) did not achieve MPR.

**Table 2:** Clincal demograchics of EGFR-mutant patients regarding different treatment modalities.

Characteristics	All (*N* = 50)	CTONG1103-GDLC cohort		Multi-center cohorts	*P*-value
		GC (*n* = 15)	Erlotinib (*n* = 16)	IO + CT (*n* = 19)	
Age at diagnosis (y), mean (SD)	60.2 (10.2)	61.1 (11.0)	57.1 (12.5)	62.2 (9.8)	0.315 (T-test)
Gender, *n* (%)					0.775 (Chisq)
Male	18 (36.0)	5 (33.3)	5 (31.2)	8 (42.1)	
Female	32 (64.0)	10 (66.7)	11 (68.8)	11 (57.9)	
Smoking status, *n* (%)					0.247 (Fisher)
Never	40 (80.0)	14 (93.3)	11 (68.8)	15 (78.9)	
Former	10 (20.0)	1 (6.7)	5 (31.2)	4 (21.1)	
Histology, *n* (%)					0.709 (Chisq)
Adenocarcinoma	45 (90.0)	14 (93.3)	13 (81.3)	18 (94.7)	
Squamous	4 (8.0)	1 (6.7)	2 (12.5)	1 (5.3)	
Others	1 (2.0)	0 (0)	1 (6.2)	0 (0)	
TNM stage, *n* (%)					0.084 (Chisq)
II	4 (8.0)	0 (0)	0 (0)	4 (21.1)	
IIIA	34 (68.0)	9 (60.0)	13 (81.3)	12 (63.1)	
IIIB	12 (24.0)	6 (40.0)	3 (18.7)	3 (15.8)	
EGFR mutation, *n* (%)					0.082 (Fisher)
Exon 19Del	17 (34.0)	6 (40.0)	5 (31.2)	6 (31.6)	
Exon 21L858R	28 (56.0)	9 (60.0)	11 (68.8)	8 (42.1)	
Insertion	5 (10.0)	0 (0)	0 (0)	5 (26.3)	

GC Gemcitabine/cisplatin; IO + CT, PD-1/PD-L1 blockade plus platinum based chemotherapy.

### Inter-tumoral heterogeneity of immune contexture between PL and DLNs might reveal diverse response to neoadjuvant immunotherapy

**Fig. 3:**
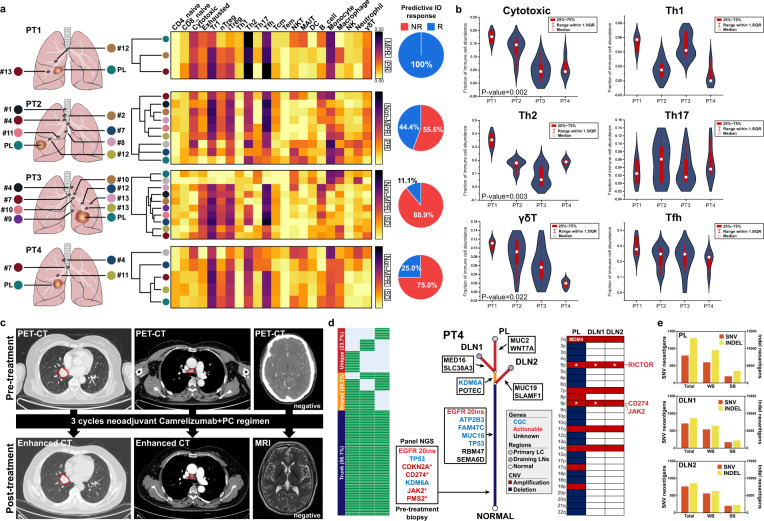
Overview of infiltrating immune contexture and genomic profile of resected PL and DLNs after neoadjuvant immunotherapy. **a** Non-supervised clustering of infiltrating immune microenviornment among PL and DLNs through ImmuCellAI. Pie chart indicates the proportion of samples which are antipated as responsive to immunotherapy. R Responder, NR Non-responder. **b** Comparison of specific immune cell subtypes among different patients. Kruskal-Wallis test was used to testify the difference among patients. Centre dots represent the median value of specific immune cell infiltration. Error bars represent the upper and lower quartiles and whiskers defines the range within 1.5IQR. **c** Radiological evaluation before and after neoadjuvant immunotherapy for PT4. **d** Genomic evolutionary trajectory between PL and DLNs of PT4. **e** Indel- and SNV-induced neoantigens burden among PL and DLNs through NeoPredPipe.

To further illustrate why localized oncogene-mutant NSCLC might be responsive or ineffective to immunotherapy, we investigated the inter-tumoral heterogeneity and immune contexture data of four patients via genomic and transcriptomic analysis of their primary lung cancer (PL) and draining lymph nodes (DLNs). We used ImmuCellAI^20^ to estimate the abundance of infiltrating immune cells and performed unsupervised-clustering. We found distinct immune microenvironment (IME) between PL and normal lymph nodes while metastatic lymph nodes showed similar IME with PLs. Interestingly, we found a pretreatment metastatic N2 lymph node from patient 2 which exhibited no residual cancer after neoadjuvant treatment showed similar IME with PL instead of other normal lymph nodes. We also applied ImmuCellAI to predict the efficacy of immunotherapy where MPR patient showed infiltrating immune features of both PL and DLNs favored immunotherapy and remarkably less tissues showed response to immunotherapy for non-responders (Fig. 3a). Relatively higher abundance of cytotoxic, Th2 and γδT cells were found in responders (Fig. 3b). For patient 4 with N2 disease, who showed completely no response to neoadjuvant immunotherapy plus chemotherapy (Fig. 3c), we established a phylogenetic tree regarding PL and metastatic lymph nodes. PL retained driver EGFR mutation and other cancer associated genes after neoadjuvant immunotherapy. Although this patient had CD274 amplification which may predict better response to immunotherapy^21^, we also identified MDM4 amplification in PL which could predict hyperprogression for immunotherapy^22^ though no MDM4 amplification was found in metastatic DLNs (Fig. 3d). Additionally, we used NeoPredPipe^23^ to evaluate neoantigen burden, and found notably higher Indel-induced neoantigens burden in PL compared to DLNs while similar for low SNV-induced neoantigens burden (Fig. 3e).

**Fig. 4:**
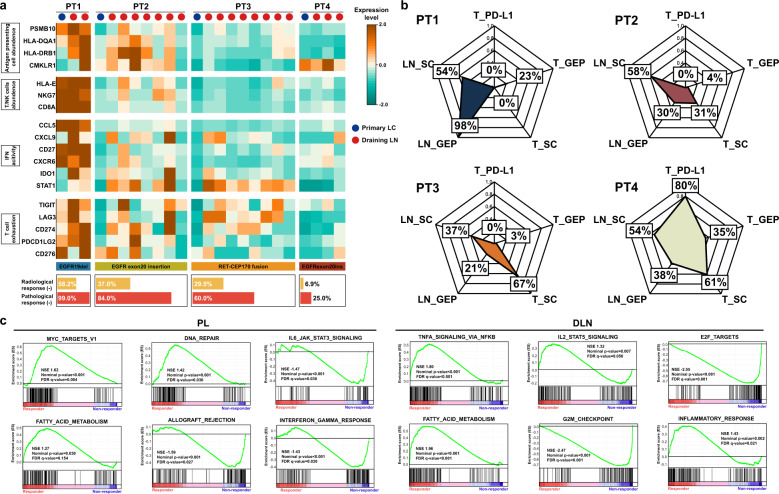
Immune phenotypes and enriched functional pathway among PLs and DLNs. **a** Expression profile of functional genesets including antigen presenting cell abundece, T/NK cell abundence, IFN activity and T cell exhasution were presented through z-score. **b** Radar plots of comprehensive immune phenotypes including PD-L1 expression in PL, T-cell inflamed GEP and suppressor cell score in PL and DLNs were scaled into 0–100%. T_PD-L1 represents expression of PD-L1 in tumor; T_GEP represents gene-expression profile of infiltrating lymphocytes in tumor; T_SC represents suppressor cell in tumor; LN_GEP represents gene-expression profile of infiltrating lymphocytes in draining lymph nodes; LN_SC represents suppressor cell in draining lymph nodes. **c** Gene set enrichment analysis (GSEA) of PL and DLNs upon responders and non-responders.

To further assess immune microenvironment phenotypes, we applied different functional gene sets upon antigen presenting cells (APC) abundance, T/NK cells abundance, IFN activity and T cell exhaustion among primary lung cancer and draining lymph nodes. Notably high expression of these gene sets was found in both PL and DLNs from responder while relatively lower expression in non-responder. Interestingly, despite low expression of these gene sets was observed in PL of PT2, relatively high expression of APC abundance and IFN activity were enriched in corresponding DLNs, and this patient only had 16% residual tumor left in the PL, which might indicate an underlying role of inflamed phenotype in DLNs for promoting the efficacy of immunotherapy (Fig. 4a). Through the radar plot, we comprehensively presented a multi-dimensional immune index regarding PD-L1 of PLs, T-cell inflamed gene-expression profile (GEP)^24^ and suppressor cell (SC)^25^ score in PLs and DLNs, respectively. We identified elevated T-cell inflamed GEP in DLNs along with lower SC score in PL for responders while the opposite in non-responders (Fig. 4b). Gene set enrichment analysis (GSEA) further showed that E2F_TARGETS, G2M_CHECKPOINT enriched in DLNs for non-responders suggesting proliferation arrested of immune cells while IL2_STAT5 and TNFA_SIGNALING for responders which are involved in anti-tumor immune response. For PLs, DNA_REPAIR and FATTY_ACID_METABOLISM were notably enriched in responders while enriched IL6_JAK_STAT3, an immune-suppression pathway, was found in non-responders (Fig. 4c).

## Discussion

Despite being a retrospective study with a heterogeneous population, our study described the clinical efficacy of neoadjuvant immunotherapy in the largest cohort of patients with resectable NSCLC harboring oncogene mutations. We observed both encouraging MPR and pCR rate for oncogene-mutant NSCLC treated with neoadjuvant immunotherapy especially for EGFR-mutant NSCLC as well as well-tolerated safety profile.

Recently released data from Checkmate-816^26^ showed superior pathological response of neoadjuvant immunotherapy plus chemotherapy compared to chemotherapy alone^27^. However, most of the neoadjuvant immunotherapy trials precluded *EGFR/ALK* mutant patients due to the inferior efficacy of immunotherapy in advanced NSCLC harboring *EGFR/ALK* mutation^4,6,8,28^. In addition, rare oncogenic mutations were reported to be either insensitive or undetermined efficacy to immunotherapy. Subgroup analysis of NEOSTAR study suggested less efficacy of either neoadjuvant nivolumab or nivolumab plus ipilimumab in canonical oncodriver patients^29^. However, phase 2 trials evaluating neoadjuvant immunotherapy plus chemotherapy^16,30^ showed astonishing pathological response in EGFR-mutant NSCLC, although both contained quite a small sample. In our study, we found that the short-term use of neoadjuvant immunotherapy plus chemotherapy in oncogene-mutant NSCLC could still achieve 37.5% MPR generally which were similar to Checkmate-816 suggesting clinical potentials for neoadjuvant immunotherapy used for oncogene mutant NSCLC. Previous study of neoadjuvant EGFR-TKIs in EGFR mutant patients exhibited relatively lower response rate compared to first-line setting in advanced disease, and MPR was unexpectedly lower which might be due to highly selective TKIs and recurrent co-occurring insensitive mutation clones^17,31^. Interestingly, unlike advanced disease, we found comparable efficacy of neoadjuvant immunotherapy for *EGFR*-mutant patients with MPR of 42.1%. Despite a lower pCR than in Checkmate-816, 10.5% EGFR-mutant patients could still achieve pCR through neoadjuvant immunotherapy while no pCR was found in the EMERGING study^17^. To be noticed, patients with EGFR rare point mutation or exon20 insertion did not show satisfactory efficacy rates in response to neoadjuvant immunotherapy plus chemotherapy, suggesting other treatment modalities are required for these patients.

Although LCMC4, an umbrella trial evaluating corresponding TKIs for different driver mutations as neoadjuvant treatment, was about to initiated, there remains an open question as to whether neoadjuvant immunotherapy might be feasible for these rare oncogene-mutant patients in consideration of one third of these patients achieved MPR in our cohort and no available neoadjuvant targeted therapy data for these patients. Indeed, limited sample size of these patients might be underpowered to draw solid conclusions.

A number of prior studies had evaluated potential biomarkers for MPR, but no agreement was made upon predictive biomarkers currently. PD-L1 status, the most well-known predictive biomarkers of CPIs in advanced NSCLC^32–34^, did not show correlation with MPR/pCR in some trials^27,35^. In the present study, we did not observe a significant correlation between pretreatment PD-L1 status and MPR either. For CA209-159 trial, although TMB was found to be relevant to the efficacy of neoadjuvant CPI^15^, TMB measures were not available for present study since few patients were performed WES or large panel NGS.

Owing to its retrospective nature, this study had two primary limitations of this study. First of all, the sample size is relatively small with diverse oncogene mutations and PD-1 blockades involved. Nevertheless, this is so far the largest cohort of localized oncogene-mutant NSCLC treated with neoadjuvant PD-1 blockades with median follow-up over 1 year. Besides, 19 patients harboring EGFR mutations, the most common driver mutation of lung cancer, were involved which could shed light on neoadjuvant treatment for EGFR-mutant NSCLC in a way. Last but not least, the lack of central evaluation of pathological response might lead to disparity of MPR which would potentially cause overestimation of MPR. However, evaluation of pCR would be barely affected due to its definition of no residual tumor cell at all. Moreover, pCR of EGFR-mutant NSCLC treated with neoadjuvant immunotherapy plus chemotherapy was notably higher than first-generation EGFR-TKI (CTONG1103) (MPR 9.7%; pCR 0.0%) and third-generation EGFR-TKI (NEOS, 2022 ELCC) (MPR 10.7%; pCR 3.6%).

In conclusion, we found that neoadjuvant immunotherapy plus chemotherapy could be clinically valuable for *EGFR*-mutant patients and potentially be extended to other rare driver mutations. Considering the underlying influence of tumor heterogeneity to targeted therapy, relatively low pathological response of targeted therapy in neoadjuvant setting and long-term benefit of immunotherapy, we assume front-line use of neoadjuvant immunotherapy plus chemotherapy may provide higher cured potentials and long-term survival benefit. Therefore, we proposed an underlying mode of neoadjuvant immunotherapy in optimizing whole-course treatment for localized oncogene-mutant NSCLC (Supplementary Fig. 3). Indeed, for localized NSCLC, multiplex genotyping is getting increasingly pivotal to guide personalized perioperative treatment. Ongoing trials such as LCMC4 (NCT04712877), NeoADAURA (NCT04351555) and NAUTIKA1 (NCT04302025) trials are evaluating matched neoadjuvant targeted therapies regarding molecular genotyping. However, neither MPR nor pCR which may indicate curative conditions from published prospective trials of neoadjuvant targeted therapy were satisfactory till now. An ongoing phase 2 trial called Neo-DIANA (NCT04512430) would evaluate neoadjuvant atezolizumab combined with chemotherapy and bevacizumab for *EGFR*-mutated NSCLC. Matched umbrella trials evaluating neoadjuvant targeted therapies should be expected and future trials of combination immunotherapy are warranted to further clarify its clinical significance, identify potential beneficiaries and tailor more optimal treatment modalities for oncogene-mutant localized NSCLC.

## Methods

### Patients and evaluation

We initiated a national questionnaire of real-world neoadjuvant immunotherapy in resectable NSCLC across 26 cancer research centers and 8 of them reported patients with oncogenic mutations who were treated with neoadjuvant immunotherapy under the real-world circumstances including inadequate biopsy specimen for genetic testing, negative findings of driver mutation from ctDNA, or no available TKI treatment. In case of any potential selection bias, all patients were screened and collected consecutively. Oncogenic mutations included *EGFR* mutations, *KRAS* mutations, *ALK* fusions, *RET* fusions, *ROS1* fusions, the *BRAF V600E* mutation and *HER2* mutations. Additionally, we applied data of Guangdong Lung Cancer Institute cohort from CTONG-1103 trial (NCT01407822) which evaluated either neoadjuvant gemcitabine plus cisplatin or erlotinib in EGFR-mutant stage III NSCLC. Informed consent was written and received from each patient before treatment as well as informed consents for experimentation with human subjects. This study was approved by the ethical committee of Guangdong Provincial People’s Hospital (KY-Z-2021-567-03).

Patients’ demographic and clinical data were retrospectively reviewed, including clinical information, tumor histology, molecular profile, treatment modality, radiological and pathological response assessment. Genotyping assessments were performed through various approaches, including PCR, immunohistochemistry (IHC) (for ALK fusion) and large panel next-generation sequencing (NGS). Radiological response was assessed following formal Response Evaluation Criteria in Solid Tumors (RECIST) measurements. Due to the retrospective nature, pathological assessment was performed independently in the Department of Pathology of each center following recommended assessment process^36^ to evaluate residual viable tumor (RVT) except for multiple slides. MPR and pathological complete response (pCR) was defined as less than 10% viable tumor cells in primary lesions and no viable tumor cells in both primary and lympho nodes, respectively.

Expression of PD-L1 was independently scored through Dako PD-L1 22C3 (pharmDx) assay. PD-L1 expression was quantified as the proportion of PD-L1-positive tumor cells. Positive PD-L1 expression in a given specimen was defined as ≥1% for tumor cell and ≥50% for high expression. Cases with <100 total tumor cells for scoring were defined as not applicable (NA).

### Comprehensive genomic and immune profiling of blood and tumor samples

We collected serial blood samples, post-treatment primary tumor samples, and draining lymph nodes specimens from four patients. Genomic DNA was extracted from frozen tumor and normal tissue sections using the QIAamp DNA FFPE Tissue Kit (Qiagen) following the manufacturer’s instructions. A minimum of 1ug of DNA was used for WES profiling experiment. The DNA quality was assessed by Nanodrop2000 (Thermo Fisher Scientific), and the quantity was measured by the dsDNA HS Assay Kit using Qubit 2.0 (Life Technologies). WES was only performed when the tumor proportion was above 20%. WES library was sequenced using an Illumina Novaseq 4000 platform according to the manufacturer’s instructions.

Total RNA from frozen section samples was extracted using RNeasy FFPE kit (QIAGEN). Ribosomal RNA was depleted using RNase H followed by library preparation using KAPA Stranded RNA -seq Kit with RiboErase (HMR) (KAPA Biosystems), and library quality was accessed by Agilent High Sensitivity DNA kit on Bioanalyzer 2100 (Agilent Technologies), which was then sequenced on Illumina Novaseq NGS platforms.

### Gene expression analysis and immune construction

Base calling was performed on bcl2fastq v2.16.0.10 (Illumina, Inc.) to generate sequence reads in FASTQ format (Illumina 1.8+ encoding). Quality control (QC) was performed with Trimmomatic (version 0.33). STAR (version 2.5.3a) is used for transcriptome mapping followed by isoform and gene level quantification performed by RSEM (version 1.3.0). Corresponding heatmaps were generated by in-house R scripts. To evaluate the immune construction of primary lung cancer and draining lymph nodes, we applied ImmuCellAI^20^ to comprehensively decode distribution of different infiltrating immune cells.

### Reconstruction of phylogenetic trees

SAMtools v1.3.1^37^ mpileup with parameter “-p”:20; “-P”: 20 was used to get mutation supporting reads counts across all tumor regions where a variant (SSNV or InDel) was detected in one or more regions in one patient. For those somatic variants that were not called ubiquitously across tumor regions, the missing variants were picked back up if the mutant read count was ≥3, as well as the read depth, was >10. Sites with sequencing depth ≤10 were marked as “NA”. The neighbor joining phylogenetic trees were constructed using R package named “MesKit”^38^. The recalled mutation file (.maf format) was set as input and method = “NJ” with bootstrap 100 times were set in the function “getPhyloTree”.

### Statistical analysis

Clinical and genomic data were analyzed using R Package (Version 3.3.0) or Prism 5.0 (Graph Pad Software Inc., La Jolla, CA, USA) software. Kruskal-Wallis test was applied to compare the significance among groups and Student’s *t-test* was used for comparison between groups. Correlations among different variables were examined by Pearson and presented through Pearson r and p-values. Odds ratios were used to compare pathological response between different treatment modalities and Fisher exact test was used to calculate the significance. A two-sided p < 0.05 was determined to be statistically significance.

### Reporting summary

Further information on research design is available in the Nature Research Reporting Summary linked to this article.

## Supplementary information

REPORTING SUMMARY

Supplementary file

## Data Availability

Partial clinicopathological data is presented in the manuscript. Sequential sequencing data including WES and RNA are available in the GSA-Human repository (Accession code: HRA002653). Other detailed informations are available from corresponding author (W-Z.Z., syzhongwenzhao@scut.edu.cn) of this study upon reasonable request.

## Code availability

No custom code was used in this study. WES sequencing data was analyzed using Trimmomatic, Burrows-Wheller Aligner(v0.7.12), Genome Analysis Toolkit (GATK 3.4.0) and VarScan 2. RNAseq data was analyzed using RSEM(v1.3.0), STAR(v2.5.3a), and ClusterProfiler(v3.4.4). TCR sequencing data was analyzed using Cutadapt (v1.18), PEAR (v0.9.10), and MiXCR(v2.1.11). All statistical analyses were performed in R version 3.5.0.
