# Supplementary material for: Induction immune-checkpoint inhibitors for resectable oncogene-mutant NSCLC: A multicenter pooled analysis
Source: NPJ Precis Oncol. 2022 Sep 19;6:66. doi: 10.1038/s41698-022-00301-8 (PMC9485257; doi:10.1038/s41698-022-00301-8)
Supplement: Supplementary file 1 — REPORTING SUMMARY [file 41698_2022_301_MOESM1_ESM.pdf]

nature portfolio

Corresponding author(s): Wen-Zhao Zhong

Last updated by author(s): Jun 2, 2022

Reporting Summary

Nature Portfolio wishes to improve the reproducibility of the work that we publish. This form provides structure for consistency and transparency in reporting. For further information on Nature Portfolio policies, see our Editorial Policies and the Editorial Policy Checklist.

Statistics

For all statistical analyses, confirm that the following items are present in the figure legend, table legend, main text, or Methods section.

n/a	Confirmed	
☐	☒	The exact sample size (*n*) for each experimental group/condition, given as a discrete number and unit of measurement
☒	☐	A statement on whether measurements were taken from distinct samples or whether the same sample was measured repeatedly
☐	☒	The statistical test(s) used AND whether they are one- or two-sided<br>*Only common tests should be described solely by name; describe more complex techniques in the Methods section.*
☐	☒	A description of all covariates tested
☒	☐	A description of any assumptions or corrections, such as tests of normality and adjustment for multiple comparisons
☐	☒	A full description of the statistical parameters including central tendency (e.g. means) or other basic estimates (e.g. regression coefficient) AND variation (e.g. standard deviation) or associated estimates of uncertainty (e.g. confidence intervals)
☐	☒	For null hypothesis testing, the test statistic (e.g. *F*, *t*, *r*) with confidence intervals, effect sizes, degrees of freedom and *P* value noted<br>*Give P values as exact values whenever suitable.*
☒	☐	For Bayesian analysis, information on the choice of priors and Markov chain Monte Carlo settings
☒	☐	For hierarchical and complex designs, identification of the appropriate level for tests and full reporting of outcomes
☒	☐	Estimates of effect sizes (e.g. Cohen's *d*, Pearson's *r*), indicating how they were calculated

Our web collection on statistics for biologists contains articles on many of the points above.

Software and code

Policy information about availability of computer code

Data collection	No software was used for data collection.
Data analysis	WES sequencing data was analyzed using Trimmomatic, Burrows-Wheller Aligner(v0.7.12), Genome Analysis Toolkit (GATK 3.4.0) and VarScan 2. RNAseq data was analyzed using RSEM(v1.3.0), STAR(v2.5.3a), and ClusterProfiler(v3.4.4). TCR sequencing data was analyzed using Cutadapt (v1.18), PEAR(v0.9.10), and MiXCR(v2.1.11). All statistical analyses were performed in R version 3.5.0.

For manuscripts utilizing custom algorithms or software that are central to the research but not yet described in published literature, software must be made available to editors and reviewers. We strongly encourage code deposition in a community repository (e.g. GitHub). See the Nature Portfolio guidelines for submitting code & software for further information.

Data

Policy information about availability of data

All manuscripts must include a data availability statement. This statement should provide the following information, where applicable:

- Accession codes, unique identifiers, or web links for publicly available datasets
- A description of any restrictions on data availability
- For clinical datasets or third party data, please ensure that the statement adheres to our policy

Partial clinicopathological data is presented in the manuscript. Detailed clinical data along with sequential sequencing data from several patients are available from corresponding author (Wen-zhao Zhong, syzhongwenzhao@scut.edu.cn) of this study upon reasonable request.

Human research participants

Policy information about studies involving human research participants and Sex and Gender in Research.

Reporting on sex and gender	Sex and gender were not specifically considered in this study. Given that previous studies showed female and non-smoker patients would have higher incidence of oncogene mutations, it would be expected to see more female patients.
Population characteristics	Patients with potentially resectable disease harboring oncogenic mutations included EGFR mutations, KRAS mutations, ALK fusions, RET fusions, ROS1 fusions, the BRAF V600E mutation and HER2 mutations and treated with induction immunotherapy with or without chemotherapy were consecutively collected.
Recruitment	Patients were retrospectively reviewed and those who met the inclusion criteria were collected for subsequent analysis.
Ethics oversight	Ethical committee of Guangdong Provincial People's Hospital (KY-Z-2021-567-03)

Note that full information on the approval of the study protocol must also be provided in the manuscript.

Field-specific reporting

Please select the one below that is the best fit for your research. If you are not sure, read the appropriate sections before making your selection.

☒ Life sciences ☐ Behavioural & social sciences ☐ Ecological, evolutionary & environmental sciences

For a reference copy of the document with all sections, see nature.com/documents/nr-reporting-summary-flat.pdf

Life sciences study design

All studies must disclose on these points even when the disclosure is negative.

Sample size	Patients were consecutively collected from multi-center datasets and 40 patients were eventually eligible for subsequent analysis.
Data exclusions	No data were excluded from the analyses.
Replication	Not available to this study as this is a retrospective clinical study.
Randomization	It's not relevant to this study due to retrospective research.
Blinding	Blinding is not available as this sis a retrospective research.

Reporting for specific materials, systems and methods

We require information from authors about some types of materials, experimental systems and methods used in many studies. Here, indicate whether each material, system or method listed is relevant to your study. If you are not sure if a list item applies to your research, read the appropriate section before selecting a response.

Materials & experimental systems

n/a	Involved in the study	
☐	☒	Antibodies
☒	☐	Eukaryotic cell lines
☒	☐	Palaeontology and archaeology
☒	☐	Animals and other organisms
☒	☐	Clinical data
☒	☐	Dual use research of concern

Methods

n/a	Involved in the study	
☒	☐	ChIP-seq
☒	☐	Flow cytometry
☒	☐	MRI-based neuroimaging

Antibodies

Antibodies used	PD-L1 22C3 (pharmDx)
Validation	PD-L1 expression was quantified as the proportion of PD-L1-positive tumor cells. Positive PD-L1 expression in a given specimen was defined as ≥1% for tumor cell and ≥50% for high expression. Cases with <100 total tumor cells for scoring were defined as not applicable (NA). Positive and negative control was set for each samples.
