# Supplementary material for: Induction immune-checkpoint inhibitors for resectable oncogene-mutant NSCLC: A multicenter pooled analysis
Source: NPJ Precis Oncol. 2022 Sep 19;6:66. doi: 10.1038/s41698-022-00301-8 (PMC9485257; doi:10.1038/s41698-022-00301-8)
Supplement: Supplementary file 2 — Supplementary file [file 41698_2022_301_MOESM2_ESM.pdf]

Supplementary Table 1. Corresponding molecular features, AEs profile and physcians' considerations for neoadjuvant immunotherapy.

Patient	Histology	cTNM	Molecular feature	Detective Method	Regimens	Reasons for NeoIO	AE profile during NeoTx
1	LUAD	cT4N0M0 (stage IIIA)	EGFR exon17-25 insertion	NGS	Pembrolizumab+AP (2 cycles)	Preoperative PCR/IHC negative	NR
2	LUAD	cT3N0M0 (stage IIB)	EGFR exon19del	PCR	Pembrolizumab+AP (2 cycles)	Inadequate biopsy samples for NGS	grade 1 nausea； grade 1 neutrophil count decreased; grade 1white blood cell decreased
3	LUAD	cT2aN0M (stage IIA)	ALK rearrangement	IHC	Nivolumab+AP (2 cycles)	Inadequate biopsy samples for NGS	NA
4	LUAD	cT3N0M0 (stage IIB)	HER2 exon20 insertion	PCR	Toripalimab+AP (3 cycles)	Inadequate biopsy samples for NGS, ctDNA negative	grade 1 constipation；grade 2 white blood cell decreased; grade 1 neutrophil count decreased; grade 1 anemia； grade 1 creatinine increased
5	LUAD	cT2bN0M0 (stage IIA)	EGFR exon21L858R	NGS	Nivolumab+PC (3 cycles)	Inadequate biopsy samples for NGS, ctDNA negative	grade 1 chest pain
6	LUAD	cT2aN2M0 (stage IIIA)	EGFR exon20 insertion/TP53/CD274 Amp	NGS	Camrelizumab+PC (3 cycles)	No approved corresponding TKI, high PD-L1	grade 3 anemia；
7	LUAD	cT1bN2M0 (stage IIIA)	KRAS G12D/SETD2/CREBBP/SMAD4	NGS	Pembrolizumab (2 cycles)	NA	grade 2 rash acneiform；grade 1 diarrhea
8	LUAD	cT4N0M0 (stage IIIA)	KRAS G12D	NGS	Nivolumab+PC (4 cycles)	NA	grade 2 alanine aminotransferase increased; grade 2 alopecia; grade 2 rash acneiform; grade 2 hyperthyroidism
9	LUAD	cT4N1M0 (stage IIIA)	RET-CEP170/NTRK3_G727	NGS	Nivolumab+PC (3 cycles)	No approved corresponding TKI	grade1 diarrhea; grade 2 lymphocyte count decreased; grade 1 white blood cell decreased
10	LUAD	cT2aN2M0 (stage IIIA)	LOC105376360-RET/TP53/BRCA2	NGS	Camrelizumab (3 cycles)	No approved corresponding TKI, high PD-L1	grade 1 abdominal pain; grade 1 rash acneiform; grade 1 reactive cutaneous capillary endothelial proliferation
11	Pleomorphic carcinoma	cT1cN2M0 (stage IIIA)	EGFR exon20in/CDKN2A copy number loss/TP53 /BRCA1/BRD4 /PARP1 /RAD50 /VEGFA	NGS	Nivolumab+PC (3 cycles)	No approved corresponding TKI	grade 3 anemia
12	LUAD	cT1bN2M0 (stage IIIA)	RET/CTNNB1/LRP1B/PTEN loss/ERBB3/TP53	NGS	Nivolumab+PC (3 cycles)	No approved corresponding TKI	grade 2 neutrophil count decreased
13	LUAD	cT3N0M0 (stage IIB)	KRAS G12D/TP53/MSH2	NGS	Pembrolizumab+AP (3 cycles)	NA	grade 1 lymphocyte count decreased；
14	LUAD	cT1cN2M0 (stage IIIA)	EGFR exon21/KRAS G13D	NGS	Pembrolizumab (4 cycles)	Multiple primary lung cancer and wild type for biopsy sample	grade 2 dry skin
15	LUAD	cT2bN0M0 (stage IIA)	EGFRexon19del/CCNE1/DLL3/KMT2A/AXL/NOTCH2/TP53	NGS	Nivolumab+PC (3 cycles)	Multiple co-occuring mutational background	grade 2 alopecia； grade 1 nausea
16	LUAD	cT1bN3M0 (stage IIIC)	BRAF V600E	NGS	Sintilimab+AP (4 cycles)	No approved corresponding TKI	NA
17	LUAD	cT3N0M0 (stage IIB)	KRAS G12C/TP53/FGFR2/NTRK1 fusion/MET/NTRK3	NGS	Camrelizumab+AP (4 cycles)	NTRK fusion along with KRAS G12C	grade 1 rash acneiform
18	LUAD	cT1bN1M0 (stage IIB)	KRAS G12V	NGS	Nivolumab+PP (3 cycles)	NA	anemia grade 2
19	LUAD	cT1cN2M0 (stage IIIA)	KRAS G12D	NGS	Nivolumab+PC (2 cycles)	NA	NR
20	LUAD	cT3N1M0 (stage IIIA)	KRAS exon2	PCR	Nivolumab+PC (2 cycles)	NA	alanine aminotransferase increased grade 1
21	LUAD	cT3N0M0 (stage IIB)	KRAS G12C	NGS	Nivolumab+PC (3 cycles)	NA	NR
22	LUAD	cT4N0M1b (stage IVB)	HER2 Exon20ins	PCR	Sintilimab+AP (3 cycles)	No approved corresponding TKI, high PD-L1	alanine aminotransferase increased grade 2；

23	LUAD	cT3N0M0 (stage IIB)	EGFR-L858R	NGS	Sintilimab+PC (2 cycles)	NGS performed after surgery	NR
24	LUAD	cT3N1M0 (stage IIIA)	EGFR-19del	NGS	Sintilimab+PC (2 cycles)	NGS performed after surgery	NR
25	LUAD	cT3N1M0 (stage IIIA)	CD74-ROS1fusion	NGS	Sintilimab+PC (2 cycles)	NGS performed after surgery	NR
26	LUSC	cT4N1M0 (stage IIIA)	EGFR-L858R	NGS	Sintilimab+PC (2 cycles)	NGS performed after surgery	NR
27	LUAD	cT3N1M0 (stage IIIA)	EGFR-L858R	NGS	Sintilimab+PC (2 cycles)	NGS performed after surgery	NR
28	LUAD	cT4N0M0 (stage IIIA)	EGFR exon21 L858R/L861Q	PCR	Camrelizumab+PC (3 cycles)	Neoadjuvant treatment was performed before PCR result	grade 1 reactive cutaneous capillary endothelial proliferation
29	LUAD	cT4N2M0 (stage IIIB)	KRAS G12C	NGS	Nivolumab+AP (3 cycles)	NA	NR
30	LUAD	cT3N2M0 (stage IIIB)	EGFR exon21 L858R	PCR	Sintilimab+AP (3 cycles)	Neoadjuvant treatment was performed before PCR result	grade 1 rash acneiform; grade 1 white blood cell decreased
31	LCC	cT3N2M0 (stage IIIB)	ALK fusion（IHC）	IHC	Pembrolizumab+PC (2 cycles)	Inadequate biopsy samples for NGS	NA
32	LUAD	cT2bN2M0 (stage IIIA)	EGFR	NGS	Nivolumab+AP (2 cycles)	Neoadjuvant treatment was performed before NGS result	NR
33	LUAD	cT2bN0M0 (stage IIA)	EGFR exon20ins	NGS	Sintilimab+PC (2 cycles)	Inadequate biopsy samples for NGS	NR
34	LUAD	cT3N1M0 (stage IIIA)	EGFR-L858R	NGS	Sintilimab+PC (2 cycles)	Inadequate biopsy samples for NGS	NR
35	LUAD	cT4N0M0 (stage IIIA)	EGFR exon21 L858R	NGS	Nivolumab+AP (3 cycles)	Inadequate biopsy samples for NGS	grade 1 chest pain
36	LUAD	cT2bN3M0 (stage IIIB)	HER2 exon20ins	NGS	Pembrolizumab+PC (3 cycles)	No approved corresponding TKI	grade 2 Alanine aminotransferase increased
37	LUAD	cT2aN2M0 (stage IIIA)	EGFR exon19ins	NGS	Nivolumab+AP (3 cycles)	Unknown efficacy for TKIs	grade 2 alopecia; grade 2 alanine aminotransferase increased
38	LUAD	cT1cN2M0 (stage IIIA)	SDC4-ROS1 fusion/TP53/B2M amp	NGS	Nivolumab+AP (3 cycles)	Neoadjuvant treatment was performed before NGS result	grade 1 alopecia; grade 2 alanine aminotransferase increased
39	ASC	cT4N2M0 (stage IIIB)	EGFR exon19del	NGS	Sintilimab+PC (2 cycles)	Mixed pathological subtypes	grade 1 Pruritus; grade 1 vomiting
40	LUAD	cT3N2M0 (stage IIIB)	EGFR exon19del	NGS	Sintilimab+PC (2 cycles)	PD-L1 high expression with suspected squamous component	grade 1 alopecia; grade 1 nausea;

LUAD, lung adenocarcinoma; LUSC, lung squamous cell carcinoma; ASC, adenosquamous carcinoma; LCC, large cell carcinoma; NA, not applicable; NR, not reported.

Supplementary Figure 1. Disease-free survival (DFS) of all patients and EGFR-mutant patients regarding MPR status.

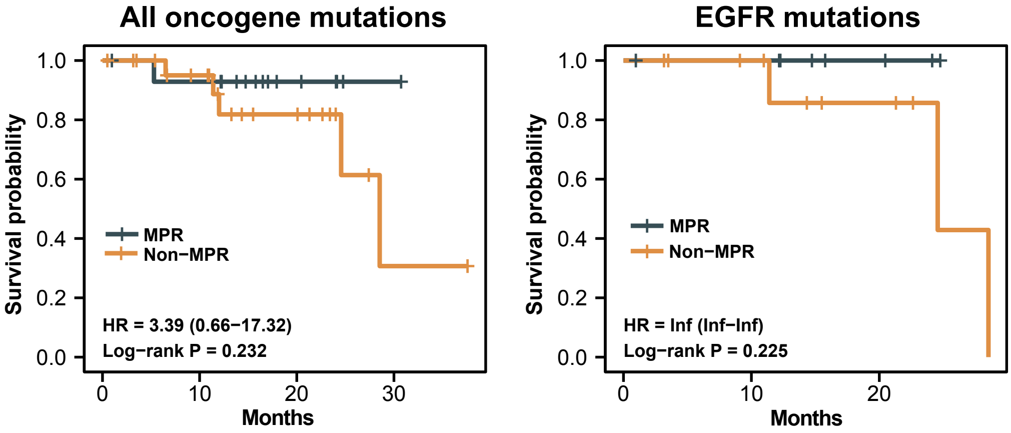

Supplementary Figure 2. Indirect comparison of major pathological response and complete pathological response between neoadjuvant immunotherapy plus chemotherapy and neoadjuvant erlotinib.

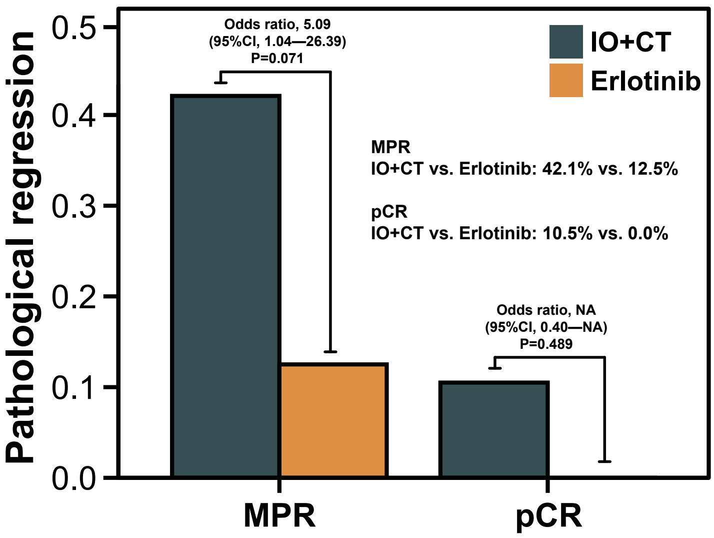

Supplementary Figure 3. Hypothesized mode of neoadjuvant immunotherapy in optimizing whole-course management for oncogene-mutant localized NSCLC.

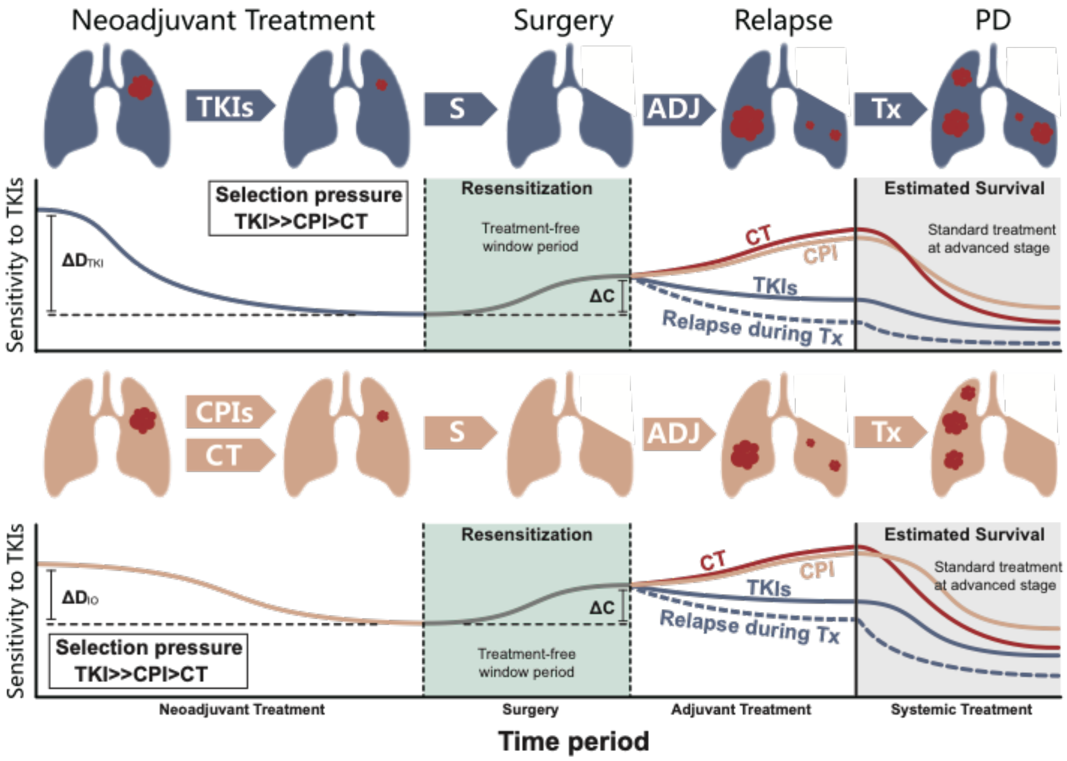

Ordinate represents sensitivity to corresponding TKIs and abscissa represents different time period starting from localized NSCLC. ΔD_{TKI} and ΔD_{IO} represents decreased sensitivity to TKIs after TKI and immunotherapy treatment, respectively. ΔC represents increased sensitivity to TKIs during treatment-free period. Survival curves during systemic treatment are based on standard treatment and not limited to single treatment modality.
